# Niches, interspecific associations, and community stability of main understory regeneration species after understory removal in temperate forests

**DOI:** 10.3389/fpls.2024.1371898

**Published:** 2024-08-29

**Authors:** Yanyan Zhang, Wangming Zhou, Quan Yuan, Jiaojiao Deng, Li Zhou, Dapao Yu

**Affiliations:** ^1^ Key Laboratory of Forest Ecology and Silviculture, Institute of Applied Ecology, Chinese Academy of Sciences, Shenyang, China; ^2^ Jilin Changbai Mountain West Slope National Research Station of Forest Ecosystem, Shenyang, China; ^3^ School of Life Sciences, Anqing Normal University, Anqing, China; ^4^ Yancheng Wetland and Natural World Heritage Conservation and Management Center, Yancheng, China

**Keywords:** understory vegetation restoration, interspecific association, species co-existence, understory removal management, community stability

## Abstract

**Introduction:**

Understory removal is frequently used to relieve the renewal pressure on trees and promote the growth capability of trees for maintaining community stability, while the lack of previous study on temperate forests limits our assessment of the effectiveness of this essential management measurement.

**Methods:**

In this study, we calculated the niche characteristics and interspecific association of main understory species and community stability in temperate forests [original broad-leaved Korean pine forest (BKF), *Betula platyphylla* secondary forest (BF), and *Larix gmelinii* plantation (LF)] after understory removal for characterizing the resource utilization capacity of the regeneration trees.

**Results:**

During the restoration stage, the niche breadth of understory plants with similar habits varied across stands and layers; regeneration tree species with heliophile and semishade occupied a larger niche in BKF and LF, while it was the opposite in LF. Niche overlap among heliophile regeneration trees increased in both BKF and BF, but not in LF. The interspecific association among main species revealed that the distribution of each species was independent and the interspecific association was loose and it varied in different forests and different light-demanding species with regeneration trees. The stability of shrub communities in BF and LF improved whereas that of BKF declined, while that of the herb communities of corresponding forests showed the opposite state.

**Discussion:**

Our study demonstrated that the effectiveness of understory removal depends on species’ ecological habits, which enhances the renewal and resource utilization capacity of regeneration tree species in temperate forests and shrub community stability in BF and LF.

## 1 Introduction

Understory vegetation is an essential component in forest ecosystems and is of great significance in maintaining community stability and regulating forest ecosystem structure and function, as well as providing habitats for animals (Nilsson and Wardle, 2005; Hart and Chen, 2008; Deng et al., 2023a). However, when the understory vegetation covers more forest land area, it has a detrimental impact on sharing environment resources with upper trees (De Lombaerde et al., 2020), slowing nutrient turnover (Wan et al., 2021), and may even trigger forest fires (Jimenez et al., 2015). Given the extensive understory vegetation potential negative impact on the renewal and growth of regeneration species (De Lombaerde et al., 2020), removing it has become an important management in attempts to achieve sustainable forestry (Deng et al., 2023b).

The Changbai Mountain forest area is a typical temperate forest ecosystem that is dominated by the original broad-leaved Korean pine forest, the natural secondary forest and plantation forest. The forests there have lush understory vegetation because of high precipitation and nutrient-rich soil (Wang et al., 2021; Deng et al., 2023b), which has hampered the reproduction and growth of regeneration tree species, such as some ornamental characteristics and precious Acer species (Wang et al., 2011). The regeneration tree species is essential for the development of forest plant communities and diversity protection (Lopez-Toledo et al., 2012), as it provides the next generation of overstory (canopy) trees (De Lombaerde et al., 2021); therefore, understanding their renewal is extremely significant.

Because of the implementation of the Natural Forest Protection Project in China in 2015, understory removal has become the main forest management measure in the forest area (Dai et al., 2011). Understory removal remains a common forest management practice to alleviate resource competition between understory vegetation and overstory trees for improving resource utilization efficiency (De Lombaerde et al., 2019, 2021). Removing the forest understory changes environmental conditions (e.g., temperature, humidity, and light availability) for reducing fire hazards (Forster et al., 2017; Elliott and Miniat, 2018), promoting seedling regeneration (Motsinger et al., 2010; Yildiz et al., 2011), and improving forest productivity (Camprodon and Brotons, 2006). Although some previous study have clarified that understory removal can have strong positive effects on tree regeneration across temperate forest contexts by analyzing seedling emergence, survival, and growth, the magnitude of these effects depended on overstory and understory conditions, the type of tree species regenerated (i.e., different ecological strategies) (Heinemann and Kitzberger, 2006; De Lombaerde et al., 2021), and removal interval (Deng et al., 2023a). Gaps caused by understory management in the short term (less than 5 years) may have complementary effects on understory light availability with benefits to seedling survival (Lhotka and Loewenstein, 2009), establishment, and growth (Beckage et al., 2008; Motsinger et al., 2010; Royo and Carson, 2022), while understory management has seen no change in regenerating overstory trees in the long term (more than 5 years) (Dodson et al., 2014; Maynard-Bean and Kaye, 2019). However, the dynamic changes in relationships among understory species have yet to be studied, which limits our assessment of the effectiveness of understory removal management; therefore, this understory management needs to be further explored.

To explain the relationships among species with different ecological habits in a heterogeneity environment, the niche method and interspecific association are commonly utilized for quantifying, which is of great significance for vegetation restoration research (Callaway and Walker, 1997; Brooker et al., 2008). Niche breadth and niche overlap are important facets of the niche method; the species with a larger niche breadth have more sufficient utilization of resources and stronger adaptability to the environment (Wasof et al., 2013), and species pairs with a large niche overlap have similar ecological characteristics (Pianka, 1973). Interspecific association is the spatial correlation of species distribution due to the difference of community habitat (Su et al., 2015), including the interaction between species and their coupling with the environment, which, when combined with the niche method, can reveal the community structure and dynamic changes when external conditions change or disturbance exists (Jin et al., 2022; Pandey et al., 2023). Some studies found that the closer the association between species pairs, the stronger the positive correlation when the stability of environmental conditions and the community increases, which means that community stability is related to the relationship between species (Jin et al., 2022; Wu et al., 2022). Therefore, understanding the relationships among species is crucial for the assessment of vegetation restoration and community stability, and can provide theoretical basis for forest management.

Consequently, the goal of this study was to estimate the effectiveness of understory removal in reducing the competition among understory plants with regeneration tree species, conserving and promoting the growth of regeneration tree species, and applying this measure to three typical stands (original broad-leaved Korean pine forest, *Betula platyphylla* secondary forest, and *Larix gmelinii* artificial forest). In an attempt to systematically assess the justifiability of understory removal, we used the M. Godron stability index, niche and interspecific association method, and the following questions were particularly addressed:

1) What are the changes of niche characteristics and interspecific relationships of main understory species with varying light demands, particularly regeneration trees (*Acer mandshuricum*, *Acer ukurunduense*, *Acer pseudosieboldianum*, *Acer tegmentosum*, and *Acer pictum* subsp. *mono*) in the understory vegetation restoration stage? We expected that the dominant position of heliophile species will be enhanced without surrounding vegetation as release from competition for light, nutrients, and moisture is likely to ease the relationship between understory vegetation and regeneration species
2) Is the relationship between the same species in different stands consistent in the understory vegetation restoration stage? Because the previous study demonstrated that the nutrient turnover rate (Miyamoto and Hiura, 2008) and light availability (Salemaa et al., 2023) of a broad-leaved forest were higher than that of a coniferous forest, we hypothesized that the relationship between species in the *B. platyphylla* forest will be weaker than other stands.
3) Is the stability of herb community and shrub community consistent? Removal of denser understory vegetation under open understory conditions is expected to result in stronger benefits for promoting broad-leaved forests’ shrub community stability than herb community stability.

## 2 Materials and methods

### 2.1 Study area

This study was conducted in Fusong county in Jilin Province, the northwest of Changbai Mountain (127°29′ to 128°02′E; 42°20′ to 42°40′N). The region is characterized by a north temperate continental climate with prolonged and severe winters and a hot and humid summer. The precipitation is mainly concentrated from July to August with an average annual precipitation of 800–1,040 mm, and the average annual temperature is 4.5–7.8°C (Chen et al., 2004). The forest types there include a small portion of the original broad-leaved Korean pine forest, and the majority of the natural secondary forests are restored after destruction, and the zonal vegetation is broad-leaved Korean pine forest; the main tree species are *Pinus koraiensis*, *B. platyphylla*, *L. gmelinii*, and *Acer pictum*.

### 2.2 Experimental design

In July 2015, three large monitoring plots with an area of 1 ha were set up in the original broad-leaved Korean pine forest (coniferous and broad-leaved mixed forest, BKF), natural secondary *B. platyphylla* forest (broad-leaved forest, BF), and *L. gmelinii* plantation forest (coniferous forest, LF). All large plots were replicated at least 300 m away from each other to minimize spatial autocorrelation. Understory removal in each plot was performed by a kind of machine that is used to cut the aboveground part of the understory shrubs, vines, and tall herbs that hinder the growth of seedlings, saplings, and trees. In July 2020, we randomly set up plots with similar slope positions and aspects with dimensions of 20 m × 20 m in three stands of two forest management measures (understory removal and retaining). Three replicates for each stand of each forest management measure (4 plots × 3 stands × 2 measures = 24 plots) were established. Four 1 m × 1 m subplots for the herbaceous population and four 5 m × 5 m subplots for shrubs were established in each management plot, and basic information of all understory species, including their names, abundance, height, and coverage, were investigated.

### 2.3 Niche characteristic

In this study, species with frequency >1 in understory left intact and removal plots were selected as the main species for analysis. The “Levins” method and “Pianka” method were used to calculate the niche breadth for main species and the niche overlap between the main species, respectively, and their formulas were as follows (Colwell and Futuyma, 1971):


(1) $${\text{B}}_{\text{i}}= 1/\sum_{j=1}^{\text{r}}{\text{P}}_{\text{ij}}^{2}$$

(2) $${\text{O}}_{\text{ih}}\text{ = }\sum_{j=1}^{\text{r}}{\text{P}}_{\text{ij}}{\text{P}}_{\text{hj}}/\sqrt{{\left(\sum_{j=1}^{\text{r}}{\text{P}}_{\text{ij}}\right)}^{2}\times (\sum_{j=1}^{\text{r}}{\text{P}}_{\text{hj}}{)}^{2}}$$

where *B_i_* is the niche breadth of species *i*, *O_ih_* is the niche overlap value of species *i* and species *h*, and *P_hj_* are the important values of species *i* and species *h* on treatment *j*, respectively.

### 2.4 Interspecific association

The variance ratio (*VR*) test was used to gain insight into the overall association among the different species, and significance was further tested using *W* statistics value. The formulas were listed as below (Schluter, 1984):


(3) $$P_{i}=n_{i}/N$$

(4) $${\text{VR = S}}_{\text{T}}^{2}{\text{/δ}}_{\text{T}}^{2}\text{ = [}\frac{1}{\text{N}}\sum_{\text{i}=1}^{\text{N}}{{\text{(T}}_{\text{j}}\text{-t)}}^{2}\text{]/}\sum_{\text{i=n}}^{\text{S}}{\text{P}}_{\text{i}}{\text{(1-P}}_{\text{i}})$$

(5) W=VR×N

where *n_i_* is the number of quadrats containing species *i*, *N* is the total number of quadrats, *S* is the total number of species, *T_j_* is the number of species occurring in quadrat *j*, and *t* is the average number of species in the quadrats. If *VR* > 1, species have a positive association. Species show a negative association when *VR* < 1. *VR* = 1 indicates that species have no associations because they are assumed independent. If ${\text{χ}}_{0.95\text{(N)}}^{2}$ <*W*< ${\text{χ}}_{0.05\text{(N)}}^{2}$ , the overall interspecific association is not significant (*p* > 0.05). Conversely, the overall interspecific association is significant (*p* < 0.05) when $W<{\text{χ}}_{0.95\text{(N)}}^{2}$ or $W>{\text{χ}}_{0.05\text{(N)}}^{2}$ .

The degree of association was conducted based on a 2×2 contingency column table, which is generated by the existence or absence of the two species. *χ* ^2^ was corrected by Yates continuous correction formula since the study was a discontinuous sample, and we determined the sign of association between species pairs by the sign of the *V* value (Wang and Peng, 1985). *b* value and *d* value were weighted to 1 to avoid a noncomputable situation when the denominator was 0 and the frequency occurrence of a certain species was 100% (Wang and Peng, 1985). These were calculated as follows:


(6) $${\text{χ}}^{2}\text{ = N(}\left|\text{ad-bc}\right|-\frac{1}{2}{\text{n)}}^{2}\text{/[(a+b)(c+d)(a+c)(b+d)]}$$

(7) V = [(a+d)-(b+c)]/(a+b+c+d)

When *χ* ^2^<3.841, there is insignificant interspecific association between two species pairs (*p* > 0.05); when 3.841 ≤ *χ* ^2^ ≤ 6.635, the interspecific association between two species pairs is significant (0.01 ≤ *p* ≤ 0.05); when *χ* ^2^>6.635, the interspecific association between two species pairs is highly significant (*p* < 0.01). In addition, if *V* > 0, there is a positive association. Conversely, if *V* < 0, there is a negative association (Forbes et al., 1994).

Pearson correlation coefficient was further used to test the strength of interspecific association (Bishara and Hittner, 2012); the formula was as follows:


(8) $$r_{s(i,h)}=\frac{\sum_{j=1}^{N}\left(x_{ij}-{\bar{x}}_{i}\right)\left(x_{hj}-{\bar{x}}_{h}\right)}{\sqrt{{\sum_{j=1}^{N}{\left(x_{ij}-{\bar{x}}_{i}\right)}^{2}\sum_{j=1}^{N}\left(x_{hj}-{\bar{x}}_{h}\right)}^{2}}}$$

In the formula, ${\text{r}}_{\text{s(i, h)}}$ is the Pearson correlation coefficients between species *i* and species *h* in the *j*-square, *N* is the total number of squares, ${\text{x}}_{\text{ij}}$ and ${\text{x}}_{\text{hj}}\;$ are the abundance values of species *i* and species *h* in the *j*-square, which form the vectors ${\text{x}}_{\text{i}}$ and ${\text{x}}_{\text{h}}$ , respectively; ${\bar{x}}_{i}$ and ${\bar{x}}_{h}$ are the average abundance of species *i* and species *h* in all quadrats, respectively. The range of ${\text{r}}_{\text{s}}\text{(i, h)}$ is [−1, 1]; a positive value indicates positive correlation, and a negative value indicates negative correlation.

### 2.5 Community stability

In previous studies, the M. Godron method was confirmed to be reliable for systematically and comprehensively quantifying community stability (Jin et al., 2022; Zhang et al., 2022), and the specific calculation method is as follows (Zheng, 2000): with different management types of forests as units, the frequency of all shrub and herb species in the community is arranged in order from large to small, and the relative frequency is gradually added and accumulated, one by one corresponding to the reciprocal accumulation of the total number of species; the scatter plot and the smooth curve are used to simulate the binomial equation, and the intersection coordinates of the equation and the equation *y* = –*x* + 100 are calculated. If the coordinates are closer to the community stability point coordinates (20, 80), the stability of the community is higher.

### 2.6 Statistical analysis

The niche characteristics and association of species, Pearson correlation analysis, and plotting were realized by the packages “spaa” and “corrplot” in the RStudio software. Excel was used to calculate the M. Godron stability of the community.

## 3 Results

### 3.1 Niche breadth of main species

During the restoration stage, in BKF, heliophiles *Ps* (1.75) with the smallest niche breadth in the shrub layer transformed to the greatest (3.00), while heliophiles *Og* with the highest niche breadth (decreased from 3.87 to 1.34) in the herb layer showed the opposite tendency (**Table 1**). However, the niche breadth of heliophiles in shrub and herb layers such as *Ps* (from 2.22 to 2.97) and *Eh* (from 1.25 to 2.88) in BF all increased (**Supplementary Figure 1**). In LF, the heliophiles *Dp* (from 2.80 to 1.92) in the shrub layer and the sciophiles *Mu* (from 3.66 to 1.29) in the herb layer with the biggest niche breadth both decreased. In addition, during the restoration stage, the regeneration species, *Ap* in LF and *At* in BKF, disappeared, while *Au* (1.81) in BKF, *Am* (3.00) in LF, and *As* (2.00) in BF appeared and their niche breadth were large. In summary, during the restoration stage, the niche breadth of understory plants with similar habits varied across stands and layers, indicating that species’ ability to use environmental resources varies across forests, and the regeneration tree species with heliophile and semishade in BKF and BF appeared and occupied a larger niche, while that in LF disappeared.

**Table 1:** Niche breadth of main populations in the shrub layer and herb layer in different forests with different managements.

Layer	Ecological habit	Abbreviation	Species	BKF		BF		LF	
				CK	UR	CK	UR	CK	UR
Shrub	Sciophiles	*Am*	*Acer mandshuricum*						3.00
	Heliophile and semishade plants	*Es*	*Eleutherococcus senticosus*	1.80	1.99			1.72	1.99
	Heliophiles	*Rm*	*Ribes mandshuricum*	1.80	1.61			2.00	2.05
	Heliophiles	*Ps*	*Philadelphus schrenkii*	1.75	3.00	2.22	2.97	2.76	2.65
	Heliophiles	*Dp*	*Deutzia parviflora* var. *amurensis*	1.90	2.60	2.33	1.92	2.80	1.92
	Heliophile and semishade plants	*Dg*	*Deutzia glabrata*				1.32		
	Semishade plants	*Au*	*Acer ukurunduense*		1.81				
	Heliophiles	*Vo*	*Viburnum opulus* subsp. *calvescens*			2.67			
	Heliophiles	*Ap*	*Acer pseudosieboldianum*					2.00	
	Sciophiles	*Ev*	*Euonymus verrucosus*				1.80		
	Sciophiles	*At*	*Acer tegmentosum*	1.96					
	Heliophile and semishade plants	*As*	*Acer pictum* subsp. *mono*				2.00	2.31	
	Heliophiles and shade plants	*Ss*	*Sorbaria sorbifolia*		1.60	2.35	1.79	1.10	
Herb	Sciophiles	*Cl*	*Cardamine leucantha*		2.28	1.32	2.18	1.60	1.81
	Sciophiles	*Dc*	*Dryopteris crassirhizoma*	1.10	2.94	2.16	2.57	1.72	2.65
	Heliophiles	*Og*	*Ostericum grosseserratum*	3.87	1.34	2.22	2.33	2.21	1.56
	Sciophiles	*Aa*	*Arisaema amurense*	2.78		1.40			
	Heliophiles	*Gh*	*Galium hoffmeisteri*	1.69					
	Sciophiles	*Cp*	*Carex pilosa*	2.86	2.70	2.86	1.21	3.00	2.52
	Heliophiles	*Eh*	*Equisetum hyemale*			1.25	2.88	1.79	1.99
	Semishade plants	*In*	*Impatiens noli-tangere*	2.29	2.55	3.56	2.67		2.70
	Heliophile and semishade plants	*Fp*	*Filipendula palmata*	2.00	1.76	3.11	2.49	1.88	
	Sciophiles	*Ua*	*Urtica angustifolia*	1.15	1.60	2.79	1.34		
	Sciophiles	*Mu*	*Meehania urticifolia*	3.59	1.90	1.34	1.76	3.66	1.29

Mean ± SD; n = 3. Different uppercase letters indicate significant differences between different understory managements in the same stands (p < 0.05). BKF, mixed broadleaved–Pinus koraiensis forest; BF, Betula platyphylla forest; LF, Larix gmelinii forest. CK, understory left intact stands; UR, understory removal stands. The missing data in the table indicate that there is no corresponding species under this management. The same is true below.

### 3.2 Niche overlap among main species

The niche overlap values and its distribution patterns between the main species under the three forest stands showed (**Table 2**; **Supplementary Figures 2**, **3**) that during the restoration stage, in BKF, the proportion of niche overlap value <0.1 of the shrub layer was greatly reduced (from 20.00% to 6.67%), while the proportion of niche overlap value >0.5 of the shrub layer increased slightly (50.00% to 53.33%); the proportion of niche overlap <0.1 and >0.5 of the herb layer both decreased slightly. During the restoration stage, niche overlap among heliophiles (*Ps*, *Dp*, and *Rm*) with regeneration trees (*Au* and *At*) all increased (**Supplementary Figure 2**).

**Table 2:** The niche overlap distribution pattern of main understory species in three forest stands.

Layer	Forest type	CK				UR			
		<0.1	0.1–0.3	0.3–0.5	>0.5	<0.1	0.1–0.3	0.3–0.5	>0.5
Shrub	BKF	2 (20.00)	2 (20.00)	1 (10.00)	5 (50.00)	1 (6.67)	4 (26.67)	2 (13.33)	8 (53.33)
	BF	−	2 (33.33)	−	4 (66.67)	4 (26.67)	2 (13.33)	3 (20.00)	6 (40.00)
	LF	6 (28.57)	−	−	15 (71.43)	1 (10.00)	1 (10.00)	3 (30.00)	5 (50.00)
Herb	BKF	2 (5.56)	−	7 (19.44)	27 (75.00)	1 (3.57)	4 (14.29)	6 (21.43)	17 (60.71)
	BF	3 (6.67)	11 (24.44)	9 (20.00)	22 (48.89)	4 (11.11)	8 (22.22)	6 (16.67)	18 (50.00)
	LF	2 (9.52)	5 (23.81)	1 (4.76)	13 (61.90)	2 (9.52)	7 (33.33)	3 (14.29)	9 (42.86)

In BF, the proportion of overlap values <0.1 of the shrub layer increased significantly (0% to 26.67%), while the proportion of overlap values >0.5 of the shrub layer decreased significantly (66.67% to 40%); the proportion of overlap values <0.1 and >0.5 of the herb layer both increased slightly. During the restoration stage, the overlap degree between the regeneration tree *As* and heliophiles (*Ss, Ps*, and *Dp*) was more than 0.33, while that with sciophiles (*Dg* and *Ev*) was small (**Supplementary Figure 2**).

In LF, the proportion of overlap values <0.1 of the shrub layer decreased (28.57% to 10%), and the proportion of overlap values >0.5 of the shrub layer decreased significantly (71.43% to 50%); the proportion of overlap values <0.1 of the herb layer remained unchanged (both 9.52%), and the proportion of overlap values >0.5 of the herb layer decreased significantly (61.90% to 42.86%). During the restoration stage, the regeneration tree *AM* emerged and the overlap degree between it and heliophiles (*Es*, *Rm*, *Ps*, and *Dp*) all showed a large niche overlap, which suggested increased niche differentiation (**Supplementary Figure 2**).

### 3.3 Interspecific association among main species

During the recovery period, the overall association between the shrub and herb layers of BKF and the shrub layer of BF did not change, both of which were not significantly positively associated (**Supplementary Table 1**). The overall association of the shrub layer of LF changed from a significant positive association to an insignificant positive association, the overall association of the herb layer of BF changed from an insignificant negative association to a significant negative association, and the overall association of the herb layer of LF changed from an insignificant positive association to an insignificant negative association, indicating that the understory communities of these forests became more unstable after understory removal.

The results of the *χ* ^2^ test indicated that (**Supplementary Tables 2**, **3**; **Supplementary Figures 4**, **5**) during the restoration stage, there were no significant association species pairs (including regeneration trees) in the shrub and herb layers of BKF, BF and LF, which showed that the distribution of each species was independent and the interspecific association was loose. In addition, during the recovery period, the ratios of negative and positive correlation species pairs of the shrub layer of LF and the understory layer of BF showed an increasing trend, which meant the competition among populations within the understory vegetation community of BF and shrub community of LF intensified.

The results of Pearson correlation of the main species showed that (**Supplementary Figures 6**, **7**; **Supplementary Tables 2**, **3**) in terms of the shrub layer, before understory removal, there was one species pair (4.76%) with a distinctly significant (*p* < 0.01) and significant (*p* < 0.05) positive correlation in LF, that is, *Rm*–*Ap* (*r* = 1.00, *p* < 0.01), which showed an extremely significant positive correlation; there was one species pair (16.67%) with a significant (*p* < 0.05) negative correlation in BF, while there were no significant correlation species pairs during the restoration stage. In terms of the herb layer, before understory removal, there were two species pairs (5.56%) with a distinctly significant (*p* < 0.01) and one (2.78%) with a significant (*p* < 0.05) positive correlation in BKF, there was one species pair (2.22%) with a significant (*p* < 0.05) positive correlation in BF, and there was one species pair (4.76%) with a distinctly significant (*p* < 0.01) and significant (*p* < 0.05) positive correlation in LF; during the restoration stage, there was one species pair (3.57%) with a significant positive correlation (*p* < 0.05) and two (7.14%) with a significant negative correlation (*p* < 0.05) in BKF; there was one species pair (9.52%) with a significant negative correlation (*p* < 0.05) in LF.

During the restoration stage, the interspecific association among regeneration trees (*Au* and *At*) with heliophiles (*Ps*, *Dp*, and *Rm*) all became more closer in BKF, while that among the regeneration tree *As* with most of heliophiles (*Ps, Dp*, and *Dg*) was negative in BF. In addition, in LF, the interspecific association among the light-loving regeneration trees (*Ap* and *As*) with other heliophiles were all positive before removal, while that among the shade-tolerant regeneration tree *Am* with heliophiles was diversified after understory removal (**Supplementary Figure 6**).

### 3.4 Stability of understory plant community

After understory removal, the distances from the regression model of the shrub communities of BKF, BF and LF to the stable point (20,80) changed from 31.15, 29.86, and 29.30 to 34.22, 27.76, and 28.49, respectively, and that of herb communities changed from 27.07, 29.19, and 26.82 to 25.79, 34.41 and 35.73, respectively, which demonstrated that during the restoration stage, the stability of shrub communities in BF and LF improved whereas that of BKF declined, while that of the herb communities of corresponding forest stands of showed the opposite state (**Table 3**).

**Table 3:** Community stability analysis of the shrub layer and herb layer in three stands.

Layer	Forest type	Management type	Regression model	*R* ^2^	Intersection coordinate	Distance between intersection point and stable point
Shrub	BKF	CK	*y* = −0.0056*x* ^2^ + 1.4825*x* + 5.5556	0.996^**^	42.03/57.97	31.15
		UR	*y* = −0.0058*x* ^2^ + 1.652*x* − 5.8824	0.999^**^	44.20/55.80	34.22
	BF	CK	*y* = −0.0093*x* ^2^ + 2*x* − 7.619	0.995^**^	41.11/58.89	29.86
		UR	*y* = −0.0069*x* ^2^ + 1.5687*x* + 9.043	0.993^**^	39.63/60.37	27.76
	LF	CK	*y* = −0.0073*x* ^2^ + 1.6783*x* + 3.0449	0.996^**^	40.72/59.28	29.30
		UR	*y* = −0.0079*x* ^2^ + 1.7215*x* + 3.4769	0.992^**^	40.15/59.85	28.49
Herb	BKF	CK	*y* = −0.0092*x* ^2^ + 1.8762*x* + 1.5185	0.998^**^	39.14/60.86	27.07
		UR	*y* = −0.012*x* ^2^ + 2.3236*x* + 1.5149	0.994^**^	33.74/66.26	19.44
	BF	CK	*y* = −0.0082*x* ^2^ + 1.8209*x* − 1.1019	0.999^**^	40.64/59.36	29.19
		UR	*y* = −0.0045*x* ^2^ + 1.4881*x* − 1.4629	0.996^**^	44.33/55.67	34.41
	LF	CK	*y* = −0.0084*x* ^2^ + 1.76*x* + 5.2179	0.995^**^	38.96/61.04	26.82
		UR	*y* = −0.0033*x* ^2^ + 1.3472*x* + 0.5208	0.997^**^	45.26/54.74	35.73

^**^represents significant difference at the level of 0.01.

## 4 Discussion

### 4.1 Niche characteristics of understory species

Niche breadth and niche overlap are used to quantify the utilization and competition of environmental resources by species (Wasof et al., 2013). After understory removal, the soil moisture of BKF (coniferous and broad-leaved mixed forest) increased rapidly and was higher than that of LF (coniferous forests) (Zhang et al., 2024), and the understory of it has higher light availability than LF (Canham et al., 1994; Rodriguez-Rodriguez et al., 2023), which widened the distribution range of shrubs, particularly heliophiles like *Ps* with ecological characteristics of light preference, strong adaptability, and resource utilization ability. However, the light is intercepted by a significant number of light-demanding plants in the shrub layer, limiting the growth and distribution of heliophiles in the herb layer (Wilson, 1981; Begue et al., 1996), particularly *Og* with the largest niche breadth. Niche breadth of heliophiles increased in BF (broad-leaved forest) due to small changes in light and nutrient content during the restoration stage, whereas it decreased significantly or vanished in LF (coniferous forest), which may be related to poor soil nutrient content caused by understory removal (Zhang et al., 2024) and species’ poor adaptability. After understory removal, the canopy density decreased (Berrill et al., 2017), resulting in a significant increase in canopy throughfall (Takahashi et al., 2003); thus, the factor limiting the niche breadth of the herbaceous population was no longer soil water content, but rather soil ammonium nitrogen content (**Supplementary Figure 8**), which may be related to the accumulation of soil nutrients during the restoration stage. For the shrub layer, soil C/N has always been a factor influencing its population niche breadth.

During the restoration stage, the *Acer* trees were still in their juvenile stage, with the majority of them renewing well and occupying a large niche breadth, particularly *Am*, which might be related to its greater adaptability to the understory light environment than other regeneration species (*Au*, *At*, *Ap* and *As*) (Jiang et al., 2013). After a 5-year understory removal, we discovered that *Acer* trees distributed better in BKF and LF, grew, and restored well. Endangered trees, particularly *Au* in BKF and *At* in LF, were well updated and developed into specialist species; this is because the high canopy density and high soil moisture of BKF and LF offer *Acer* trees with the best shade-tolerant environment for survival. Although understory removal aided the growth and reproduction of these two *Acer* trees, it severely weakened the regenerative ability of other *Acer* trees. As a result, in terms of endangered species’ conservation, on the one hand, understory removal may be an interference rather than a beneficial management measure for BKF and LF (Swab et al., 2008); on the other hand, the complete habitat of endangered *Ace*r trees should be protected as much as possible, because if it is severely disturbed, *Ace*r trees with weak adaptability and reproductive capacity will be extruded out of the community (Saeki, 2007).

A species with a wide niche breadth is usually dominant (Pannek et al., 2016) and spreads widely in the community (Sheth et al., 2020), and it frequently has a significant niche overlap with other species in the community, indicating that these species pairs competed for similar resources (Gilbert et al., 1952). During the restoration stage, the proportion of niche overlap value < 0.1 in the shrub layer of BKF and LF decreased greatly, and the proportion of niche overlap value < 0.1 in the shrub layer of BF increased significantly. Before understory removal, the niche overlap values between the six species pairs of *Es*–*At*/*Es* and *Gh*–*Ua* in BKF, *Aa*–*Mu* in BF, and *Cl*–*Dc*/*Fp* in LF were 0, indicating that the ecological habits of these species pairs were different and the probability of interspecific encounter was low. The niche overlaps of *Aa*–*Cp* and *Ua–Dc* in BKF, and *Rm*–*Ap* in LF were all 1, indicating that these species had a consistent demand for environmental resources, high ecological similarity, and fierce interspecific competition (Mouillot et al., 2005). During the restoration stage, owing to the lack of canopy interception and redistribution of precipitation and light (De Lombaerde et al., 2021), the habitat conditions such as the increase of water content and light in the forest were changed (Swab et al., 2008), which promoted the rapid recovery and diffusion of light-demanding, wet-loving, and seed-transmitting species (Walters et al., 2016); thus, there were niche overlaps between species pairs. During the restoration stage, the overlap values between sciophiles shrubs (*Ps* and *Dp* in BKF, *Ss* and *Ps* in BF, and *Es* and *Rm* in LF) and semishade regeneration trees (*Au*, *Am* and *As*) in three stands were more than 0.5, which means that there was a fierce interspecific competition among them. The proportion of large overlap values decreased significantly in LF, which indicated that understory removal could release the competition between species and regeneration trees for environmental resources in LF to a certain extent, rather than BKF and BF, which is consistent with the previous studies (Zhang et al., 2006).

### 4.2 The interspecific association between the main understory species

Interspecific association reflects the interaction and mutual influence of various species in the community due to habitat differences and other species, and the overall association is one of the methods to characterize community stability (Chen et al., 2023). It is generally believed that with community succession, species tend to be positively associated in order to achieve stable coexistence (Zhang et al., 2022; Chen et al., 2023). This study found that during the restoration stage, the overall association of the understory vegetation of BKF was still not significant positive, which revealed that its understory vegetation community was loose and the community composition and structure were slightly unstable. This is due to the fact that the BKF is the top community in the Changbai Mountain forest region, and its community has a stronger anti-interference ability and is more likely to return to its original state after external interference (Zhou et al., 2012). However, for BF (secondary forests) and LF (plantations), the overall association of their understory vegetation changed from positive to negative during the recovery period, which revealed that the vegetation was in the early stage of succession and the populations were in a relatively independent state; this was related to their simple community structure, fierce competition for resources of understory vegetation (Oliveira et al., 2023), and weak interspecific associations (Su et al., 2015).

Both the *χ* ^2^ test and Pearson test showed that the number of positive species pairs in the shrub layer of BKF and LF was more than that of negative species pairs. The *χ* ^2^ test showed that the number of positive species pairs was more than the number of negative species pairs in the understory vegetation of BF and LF during the restoration stage, indicating that the results of the Pearson test were the opposite. Because the results of the Pearson test are more sensitive than those of the *χ* ^2^ test (Liu et al., 2018), the stability of understory plant community in BKF and LF in the restoration stage is higher, the dependence between populations is higher, and the suitability with the environment is higher.

In BKF, before understory removal, the positive correlations in *Dc*–*Fp* (*r* = 0.97, *p* < 0.05), *Ua* (*r* = 0.99, *p* < 0.01), and *Aa*–*Cp* (*r*=1.00, *p* < 0.01) were strong, which might have similar environmental adaptability. During the restoration stage, there was a significant negative correlation (*r* = −0.96, *p* < 0.05) in *Cl*–*Cp*, a species pair with hygrophilous characteristics, which were mutually exclusive in the process of competing for resources, and the probability of encountering with *In* with strong seed dispersal ability was high; thus, there was a significant positive correlation (*r* = 0.98, *p* < 0.05) between the species pairs. In addition, there was a significant negative correlation (*r* = −0.99, *p* < 0.05) in *In*–*Cp*, which had strong reproductive ability, in competing for a limited environment during seed dispersal. In LF, before understory removal, there was a strong positive correlation in *Rm*–*Ap* (*r* = 1.00, *p* < 0.01), and there was a significant positive correlation in *Ps*–*Dg* (*r* = 0.95, *p* < 0.05) because of their strong adaptability, and light and shade tolerance. During the restoration stage, there was a significant positive correlation in *Cl*–*Eh* (*r* = 0.96, *p* < 0.05) and *Og*–*Cl* (*r* = 0.97, *p* < 0.05) for sharing resources. Pearson correlation analysis showed that heliophile *Acer* trees (*At*, *Au*, *As*, *Ap*, and *Am*), which were endangered plants in this study area, were positively correlated with other shrubs in BKF and LF, but negatively correlated with other shrubs in BF, which showed that endangered plants recovered better in BKF and LF.

### 4.3 Stability of understory plant community

Our study showed that during the restoration stage, the stability of understory plant communities in different forest stands were different and varied in different layers, which revealed that the understory communities in three stands is in the early or middle stage of succession. BKF is the top forest community with high soil nutrient content (Chen and Li, 2003) and a complex canopy structure in the Changbai Mountain area (Chen et al., 2004). Therefore, during the restoration stage, the survival of some shrub plants is inhibited, while the hygrophilous plants with strong regeneration can make full use of environmental resources and increase the stability of the community. For secondary forests (BF) and artificial forests (LF), their community structure is simpler than that of mixed forests. At the same time, the soil nutrient content increases (Zhang et al., 2024), and the adaptable shrub plants are rapidly restored due to sufficient light and the increase in the number of shrub plants and the decrease in the light on the surface inhibit the regeneration of photophilous herbs, resulting in an increase in the stability of shrub communities and a decrease in the stability of herbaceous communities. In addition, the stability of shrub and herb communities showed an opposite trend; that is, the herb community stability corresponded to the shrub community instability in the same stand. This may be due to the fact that shrub and herb communities are in the early stage of succession and renewal, and the mutual exclusion effect is obviously greater than the mutual promotion and the result of fierce competition for limited resources between species pairs. Some studies (Wu et al., 2018) suggested that the community stability increased when the disturbance intensity changed from mild to moderate, but decreased when it turned to severe disturbance. Therefore, understory removal is a severe disturbance to the shrub community of BKF and the herb community of BF and LF, and a moderate disturbance to the herb community of BKF and the shrub community of BF and LF. That is to say, understory removal is a beneficial management to BF and LF, and may be a disturbance to BKF.

## Data Availability

The raw data supporting the conclusions of this article will be made available by the authors, without undue reservation.

## Supplementary material

Supplementary Figure 1 Changes of niche width of common species in three forest stands.

Supplementary Figure 2 Niche overlap of dominant species in shrub layer in different forests with different managements. The diagonal letters in the diagram correspond to **Supplementary Figure 1**; the circular size and color depth represent the size of the niche overlap between the species, and the deeper the color, the greater the value. The same is true below.

Supplementary Figure 3 Niche overlap of dominant species in the herb layer in different forests with different managements.

Supplementary Figure 4
*χ* ^2^ test semi-matrix of the main species in the shrub layer.

Supplementary Figure 5
*χ* ^2^ test semi-matrix of the main species in the herb layer.

Supplementary Figure 6 Pearson correlation semi-matrix of the main species in the shrub layer.

Supplementary Figure 7 Pearson correlation semi-matrix of the main species in the herb layer.

Supplementary Figure 8 Canonical correlation analysis analysis between soil chemical properties and population niche breadth.

Supplementary Table 1 The overall association of interspecific relationships.

Supplementary Table 2
*χ* ^2^ test and Pearson correlation test of the main species in the shrub layer.

Supplementary Table 3
*χ* ^2^ test and Pearson correlation test of the main species in the herb layer.
