# Supplementary material for: Niches, interspecific associations, and community stability of main understory regeneration species after understory removal in temperate forests
Source: Front Plant Sci. 2024 Aug 29;15:1371898. doi: 10.3389/fpls.2024.1371898 (PMC11390392; doi:10.3389/fpls.2024.1371898)
Supplement: Supplementary Figure 1 — Changes of niche width of common species in three forest stands. [file DataSheet1.pdf]

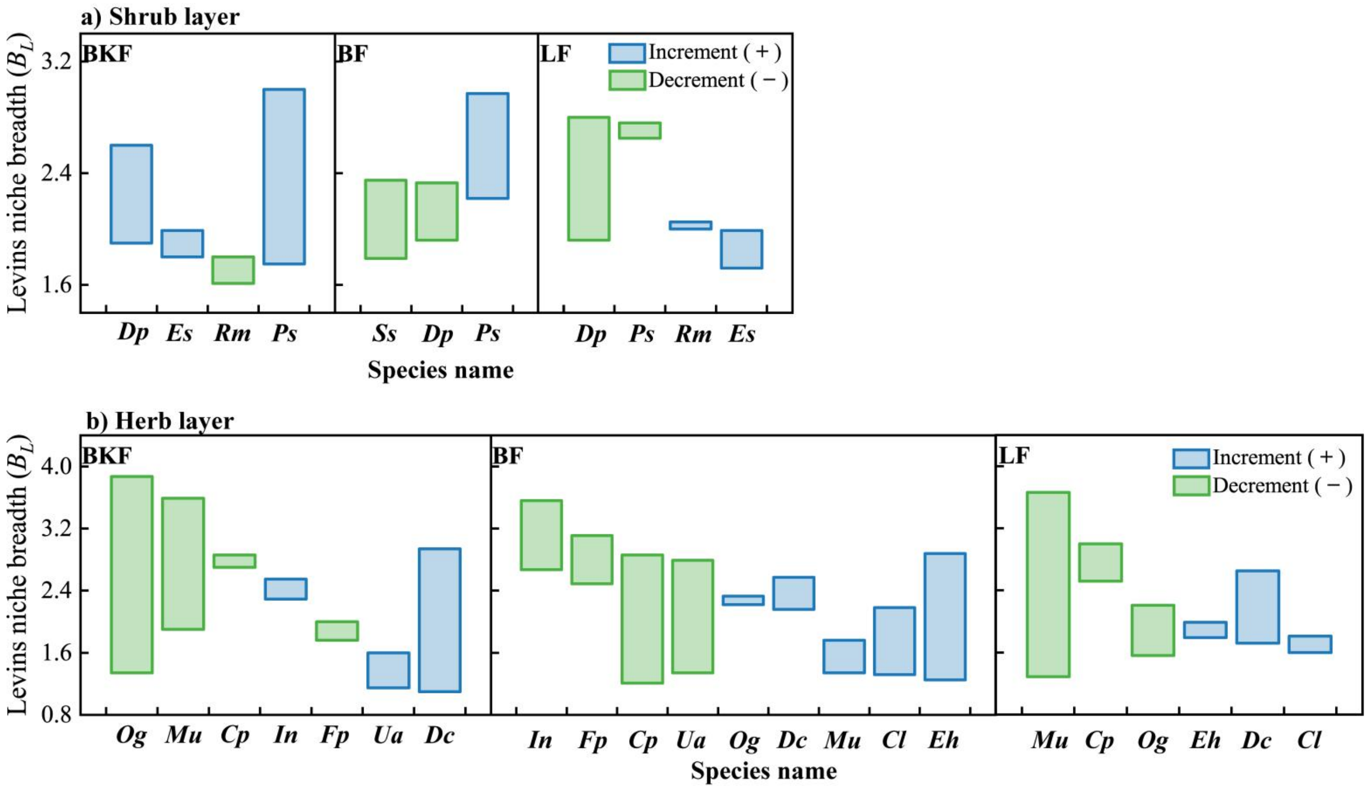

Figure S1. Changes of niche width of common species in three forest stands. BKF: mixed broadleaved-*Pinus koraiensis* forest; BF: *Betula platyphylla* forest; LF: *Larix gmelinii* forest. CK: understory left intact stands, UR: understory removal stands. Species name in the diagram corresponds to different species name in Table 1. The same as below.

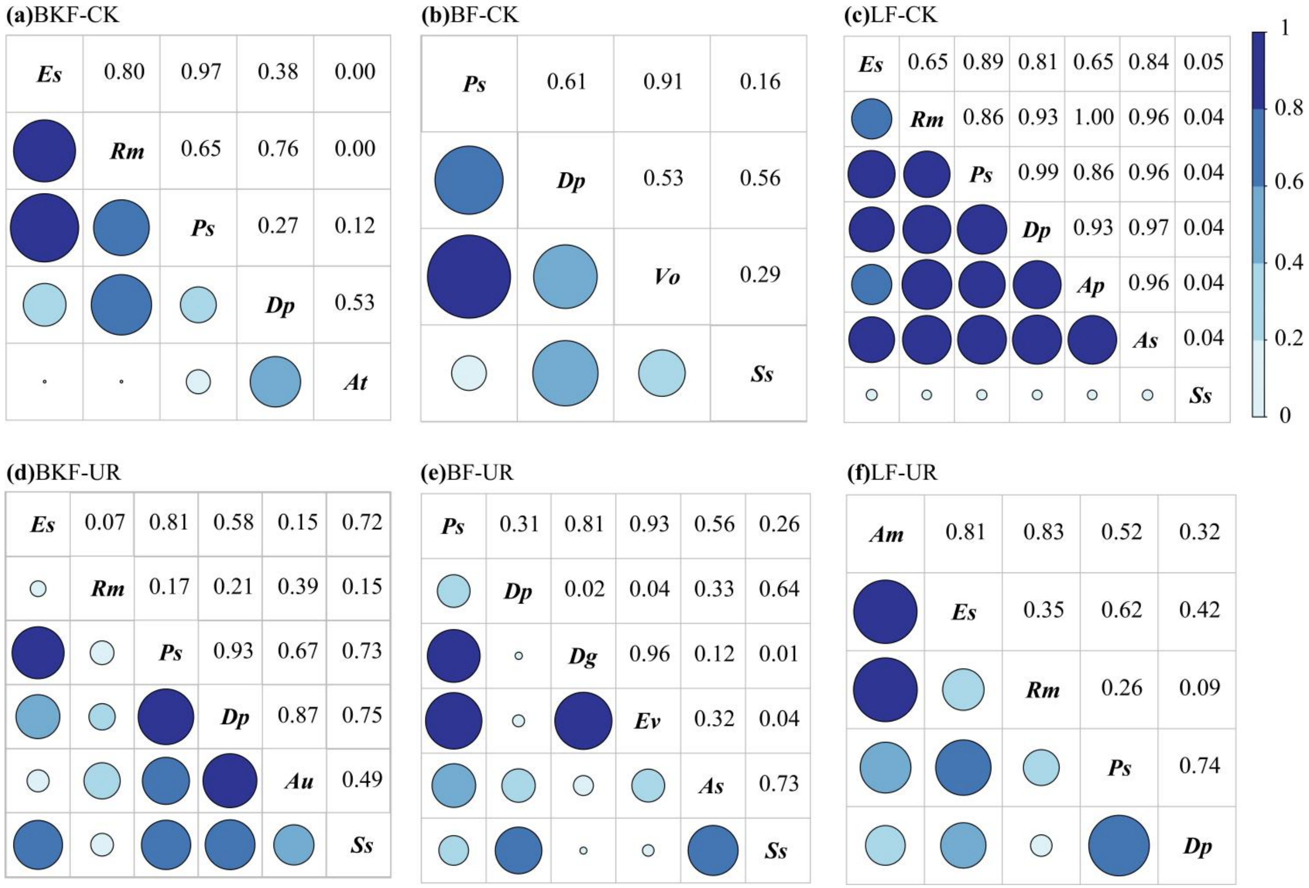

Figure S2. Niche overlap of main species in shrub layer in different forests with different managements. The diagonal letters in the diagram represents different species name in Table 1; the circular size and color depth represent the size of the niche overlap between the species, and the deeper the color, the greater the value. The same below.

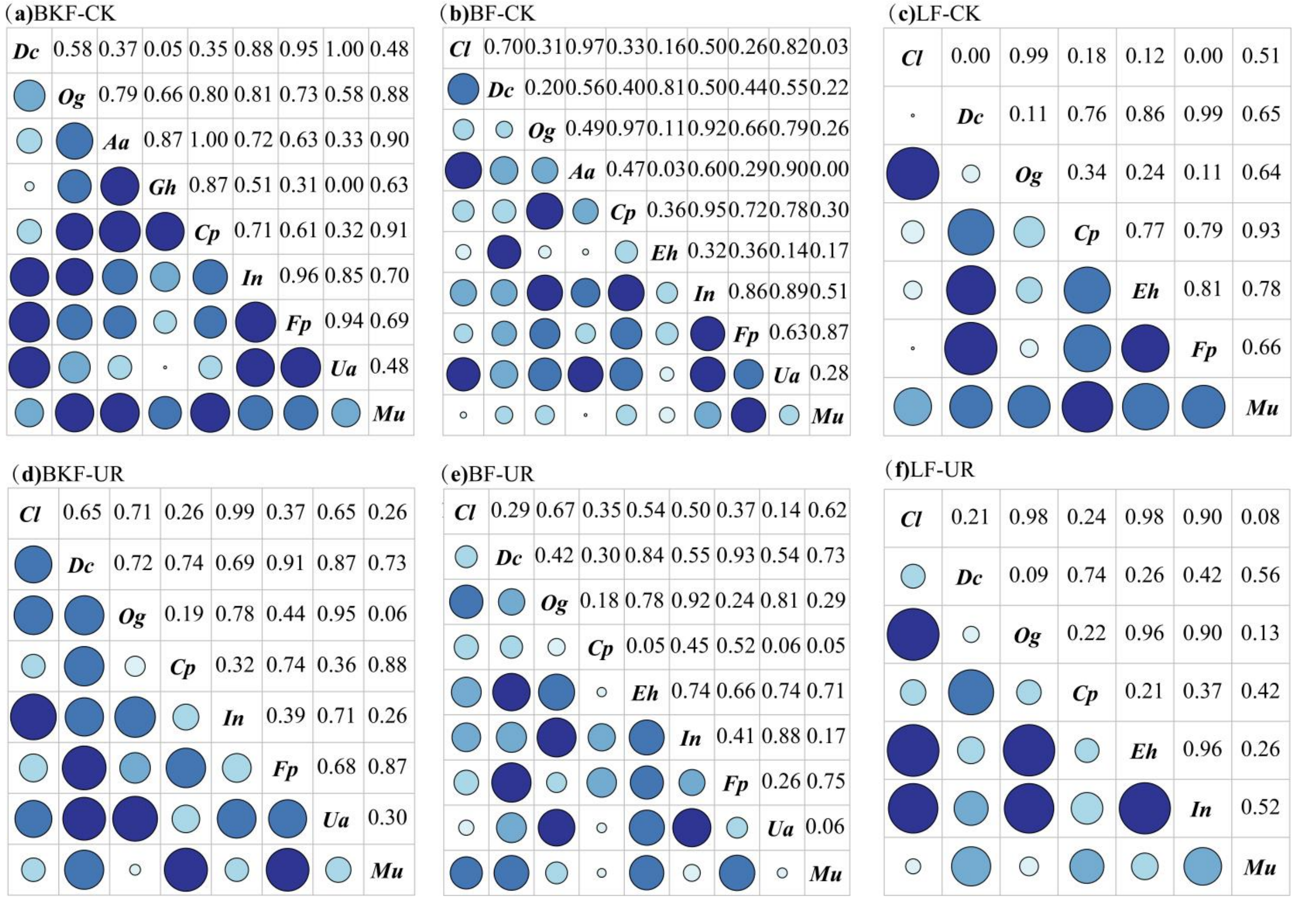

Figure S3. Niche overlap of main species in herb layer in different forests with different managements.

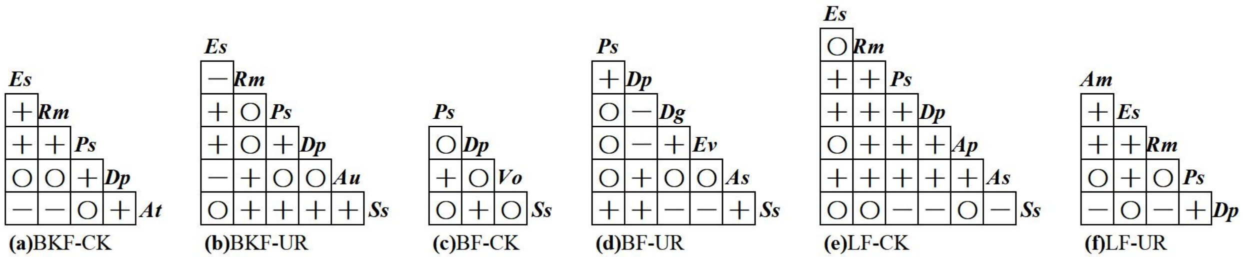

Figure S4. χ^2 test semi-matrix of main species in shrub layer. '+' indicates no significant positive association; '−' indicates no significant negative association; '○''means no association.

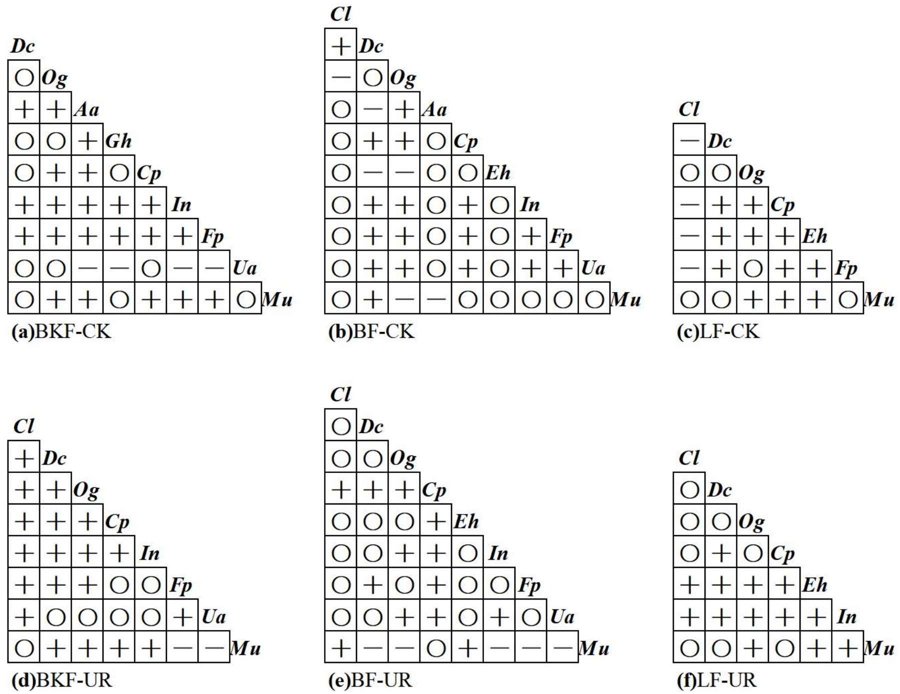

Figure S5. χ^2 test semi-matrix of main species in herb layer.

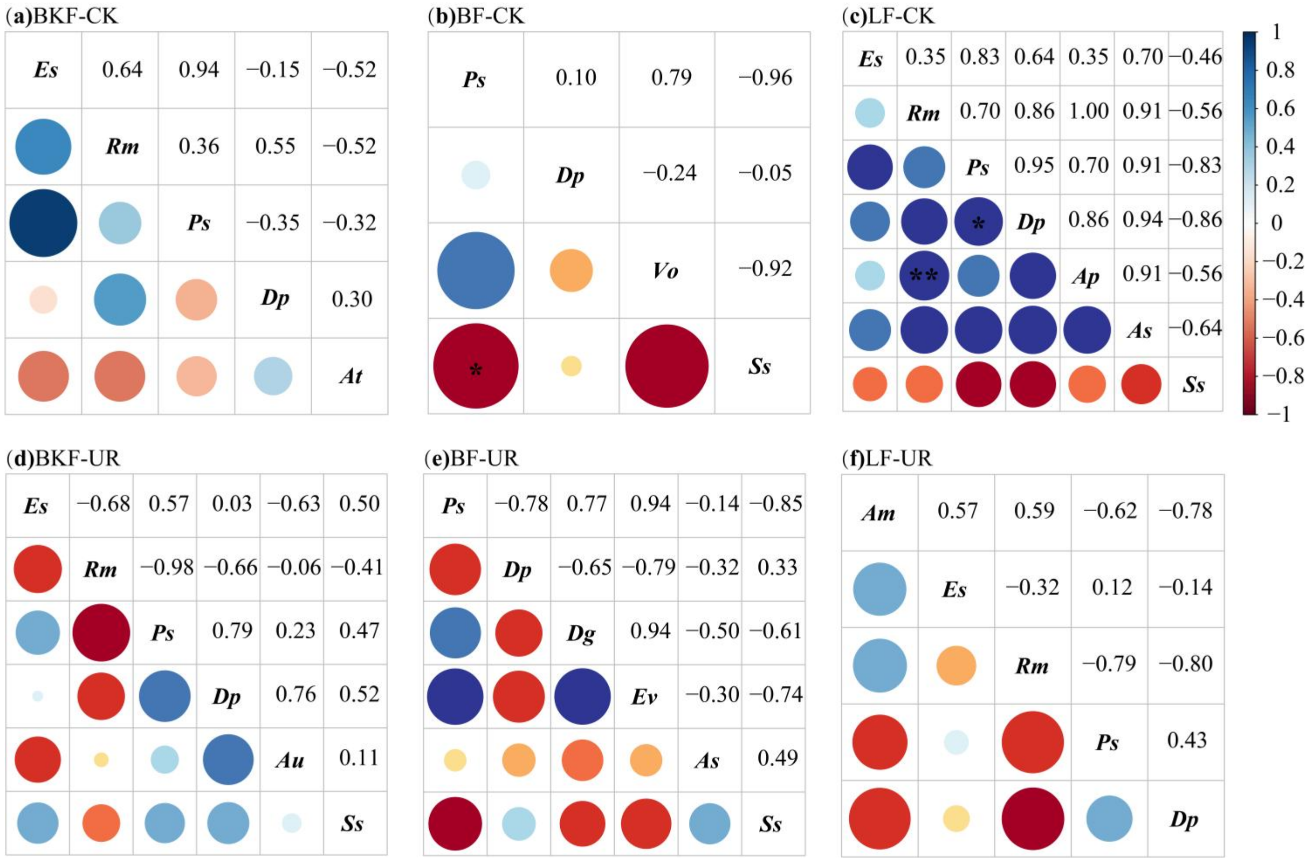

Figure S6. Pearson correlation semi-matrix of main species in shrub layer. The size of the circle and the color depth represent the Pearson correlation between the pairs, and the deeper the color, the greater the absolute value. ** $P<0.01$; * $P<0.05$; the same below.

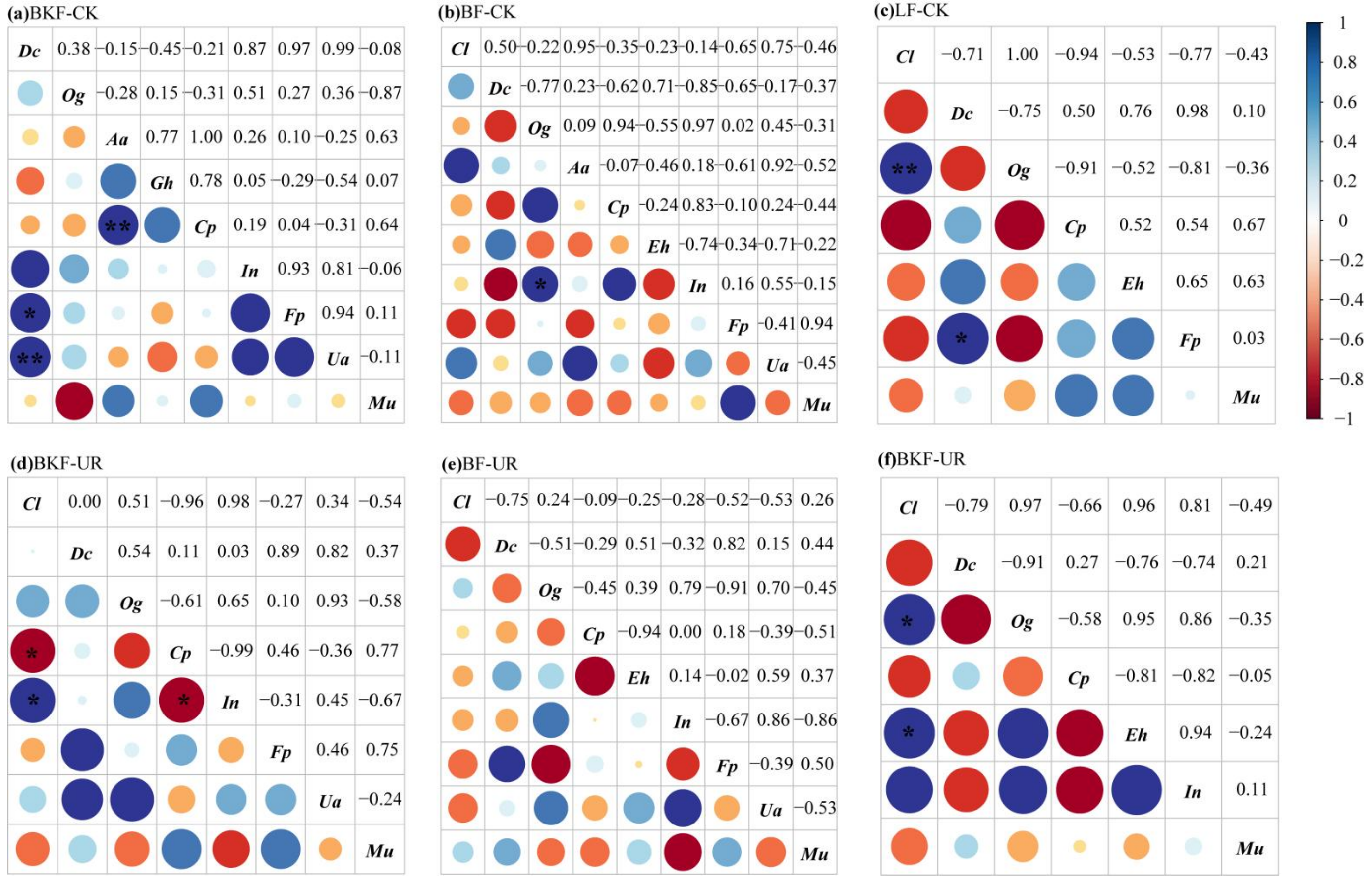

Figure S7. Pearson correlation semi-matrix of main species in herb layer.

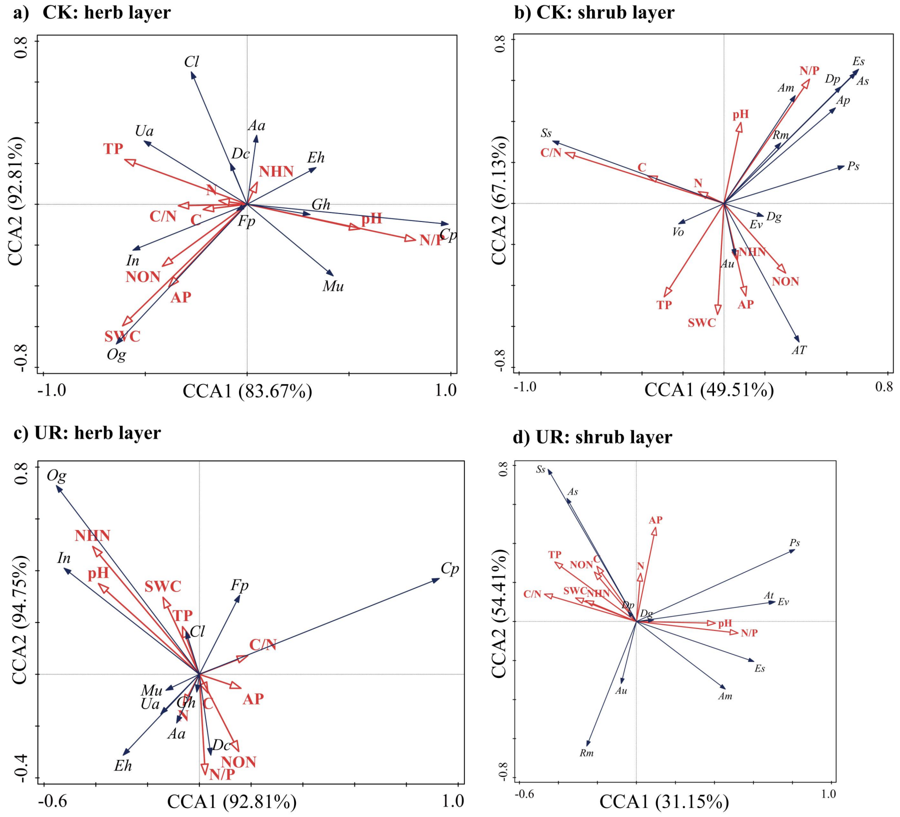

Figure S8. Canonical correlation analysis analysis between soil chemical properties and population niche breadth. (a) and (c) are canonical correlation analysis of herb species niche breadth in understory left intact (CK) and removal (UR) stands, respectively. (b) and (d) are the canonical correlation analysis of shrub species niche breadths and soil properties in left intact (CK) and removal (UR) stands. SWC, C, N, TP, AP, NHN, NON represent soil water content, total carbon (TC), total nitrogen (TN), total phosphorus (TP), available phosphorus (AP), ammonium nitrogen (NH_4^+−N) and nitrate nitrogen (NO_3^-−N) content, respectively.

Table S1. The overall association of interspecific relationships

Layer	Forest type	CK			UR		
		Variance ratio (*VR*)	Test statistics (*W*)	Test results	Variance ratio (*VR*)	Test statistics (*W*)	Test results
Shrub	BKF	2.00	8.00	Not significant positive association	2.00	8.00	Not significant positive association
	BF	1.33	5.33	Not significant positive association	1.33	5.33	Not significant positive association
	LF	3.00	12.00	Significant positive association	1.59	6.35	Not significant positive association
Herb	BKF	2.05	8.19	Not significant positive association	1.43	5.71	Not significant positive association
	BF	0.18	0.73	Not significant negative association	0.12	0.48	Significant negative association
	LF	1.33	5.33	Not significant positive association	0.73	2.93	No significant negative association

BKF: mixed broadleaved-*Pinus koraiensis* forest; BF: *Betula platyphylla* forest; LF: *Larix gmelinii* forest. CK: understory left intact stands, UR: understory removal stands. The same as below.

Table S2. χ^2 test and Pearson correlation test of main species in the shrub layer

Test type	Forest type	Management type	Positive association /correlation				Negative association /correlation				No association / correlation	The ratios of negative and positive correlations
			Distinctly significant ($P \leq 0.01$)	Significant ($P \leq 0.05$)	Not significant ($P > 0.05$)	Sum	Distinctly significant ($P \leq 0.01$)	Significant ($P \leq 0.05$)	Not significant ($P > 0.05$)	Sum		
χ^2 Test	BKF	CK	–	–	5(50.00)	5(50.00)	–	–	2(20.00)	2(20.00)	3(30.00)	0.40
		UR	–	–	8(53.33)	8(53.33)	–	–	2(13.33)	2(13.33)	5(33.33)	0.25
	BF	CK	–	–	2(33.33)	2(33.33)	–	–	-	-	4(66.67)	0.00
		UR	–	–	6(40.00)	6(40.00)	–	–	4(26.67)	4(26.67)	5(33.33)	1.50
	LF	CK	–	–	13(61.90)	13(61.90)	–	–	3(14.29)	3(14.29)	5(23.81)	0.23
		UR	–	–	5(50.00)	5(50.00)	–	–	2(20.00)	2(20.00)	3(30.00)	0.40
Pearson correlation	BKF	CK	–	–	5(50.00)	5(50.00)	–	–	5(50.00)	5(50.00)	-	1.00
		UR	–	–	9(60.00)	9(60.00)	–	–	6(40.00)	6(40.00)	-	0.67
	BF	CK	–	–	2(33.33)	2(33.33)	–	**1(16.67)**	3(50.00)	4(66.67)	-	2.00
		UR	–	–	5(33.33)	5(33.33)	–	–	10(66.67)	10(66.67)	-	2.00
	LF	CK	**1(4.76)**	**1(4.76)**	13(61.49)	15(71.43)	–	–	6(58.57)	6(28.57)	-	0.40
		UR	–	–	4(40.00)	4(40.00)	–	–	6(60.00)	6(60.00)	-	1.50

Table S3. χ^2 test and Pearson correlation test of main species in the herb layer

Test type	Forest type	Management type	Positive association /correlation				Negative association /correlation				No association/ correlation	The ratios of negative and positive correlations
			Distinctly significant ($P \leq 0.01$)	Significant ($P \leq 0.05$)	Not significant ($P > 0.05$)	Sum	Distinctly significant ($P \leq 0.01$)	Significant ($P \leq 0.05$)	Not significant ($P > 0.05$)	Sum		
χ^2 Test	BKF	CK	−	−	21(58.33)	21(58.33)	−	−	4(11.11)	4(11.11)	11(30.56)	0.19
		UR	−	−	19(67.86)	19(67.86)	−	−	2(7.14)	2(7.14)	7(25.00)	0.11
	BF	CK	−	−	17(37.78)	17(37.78)	−	−	6(13.33)	6(13.33)	22(48.89)	0.35
		UR	−	−	12(34.29)	12(34.29)	−	−	5(14.29)	5(14.29)	18(51.43)	0.42
	LF	CK	−	−	11(52.38)	11(52.38)	−	−	4(19.05)	4(19.05)	6(28.57)	0.36
		UR	−	−	13(61.90)	13(61.90)	−	−	-	-	8(38.10)	0.00
Pearson correlation	BKF	CK	**2(5.56)**	**1(2.78)**	20(55.56)	23(63.89)	−	−	13(36.11)	13(36.11)	-	0.57
		UR	−	**1(3.57)**	16(57.14)	17(60.71)	−	**2(7.14)**	8(28.57)	10(35.74)	1(3.57)	0.59
	BF	CK	−	**1(2.22)**	16(35.56)	17(37.78)	−	−	28(62.22)	28(62.22)	-	1.65
		UR	−	-	15(41.67)	15(41.67)	−	−	21(58.33)	21(58.33)	-	1.40
	LF	CK	**1(4.76)**	**1(4.76)**	9(42.86)	11(52.38)	−	−	10(47.62)	10(47.62)	-	0.91
		UR	−	−	9(42.86)	9(42.86)	**2(9.52)**	−	10(47.62)	12(57.14)	-	1.33
